# An unusual cause of constipation in a patient without any underlying disorders 

**Published:** 2015

**Authors:** Amir Sadeghi, Shabnam Shahrokh, Mohammad Reza Zali

**Affiliations:** *Gastroenterology and Liver Diseases Research Center, Shahid Beheshti University of Medical Sciences, Tehran, Iran*

**Keywords:** Constipation, Celiac, Gluten free diet

## Abstract

Constipation is the most common digestive complaint in the general population. It is associated with considerable adverse effect on quality of life and substantial economic costs and often has no underlying pathology. Non-Celiac Gluten Sensitivity (NCGS) has been described as a cause of constipation in a few studies. We present a 62-year-old male with long standing constipation without any underlying cause during extensive surveys and not responding to any conservative treatment but significant response with gluten free diet (GFD).

## Introduction

 Wheat allergy, celiac disease (CD), and Non-Celiac Gluten Sensitivity (NCGS) are three distinct conditions that are triggered by the ingestion of wheat gliadin. NCGS is an entity of Gluten-related disorders in the absence of wheat allergy or celiac disease (1-4). The pathogenesis is not well understood but the role of innate immune system and intestinal permeability are the possibilities (2). Numerous gastrointestinal and non-gastrointestinal symptoms could be developing in the setting of this clinical situation (5-7). The diagnosis is based on exclusion of CD and wheat allergy in compatible clinical setting (3). Gluten free diet is a main stay of treatment (5). A few studies have been published to date on direct effects of gluten free diet at common symptoms in gastrointestinal setting (8). In this study, we described our experience of evaluation of NCGS in a patient with long standing constipation. 

## Case Report

The patient is a 67-year-old man was seen in outpatient clinic because of a history of long standing constipation. His stool frequency was less than three per week with usually lumpy and hard stools and loose stools are rarely present without the use of laxatives. (he fulfilled ROME criteria of chronic constipation). He denied any other symptoms such as fatigue, malaise, loss of appetite, dyspepsia, abdominal pain, bloating, evidence of gastrointestinal bleeding and GERD symptoms. He did not have any past medical history and his family history was negative either. He had tried to change his diet (high fiber, high amount of fruit, vegetables with olive oil and increased consumption of daily water), osmotic agents and secretary laxatives for solving the problem, but they had not resolved his problem. His physical examination was unremarkable and his lab test which are shown in table 1, were totally normal. 

**Table 1:** The laboratory examination at the addmision

Laboratory tests	Results
WBC	4300 (/µL)
HGB	13.9 (g/dL)
MCV	83 (fL)
PLT	146000 (/µL)
ESR	3 (/1 hour)
IgA	Normal
IgE	Normal
Anti tTG Ab	Negative
Anti EMA Ab	Negative
AST	12 (IU/L)
ALT	14 (IU/L)
ALKP	146 (IU/L)
TSH	2.5 (mIU/L)
Na	143 (mmol/L)
K	4.3 (mmol/L)
FBS	83 (mg/dL)
Uric Acid	5 (mg/dL)
Stool Exam	Normal
Lipid Profile	Normal

According to his age, multiple evaluation including abdominal x-ray, endoscopy with biopsy of second portion of duodenum, colonoscopy, colon transit time, anal manometry and abdominal pelvic ultrasound were done which were totally normal. With regard to only long standing constipation and no other abnormality and disorders, gluten free diet had begun for him. He has followed his diet orders well and strictly controlled the diet. After 2 months he had defecations 4 times with normal consistency per week but after restarting of normal diet with gluten, constipation relapsed. GFD started again and after 4 months he had completely normal bowel habits.

## Discussion

Gluten, named from the latin gluten meaning glue, which is a protein composite found in wheat and related grains, including barley and rye (1) and is found mainly in foods, available food products (used as a protein filler), modified food starch, preservatives, and stabilizers made with wheat and may also be found in everyday and unexpected products, such as medicines, vitamins, and lip balms (8,9).

Gluten-related disorders has been best understood in the context of two conditions, celiac disease, a chronic small intestinal, immune-mediated, enteropathy caused by gluten ingestion and wheat allergy, an immunologic reaction which wheat specific IgE antibodies play a major role on that (10-14). However, In recent years we encountered with significant percentage of the people who report problems (both gastrointestinal and non-gastrointestinal symptoms) caused by wheat and/or gluten ingestion but the diagnosis of celiac disease (CD) or wheat allergy (WA) were ruled out by negative test for specific serology and histopathology of CD and for immunoglobulin E (IgE)-mediated assays and nearly all of them patients report improvement of symptoms on a gluten-free diet. This clinical situation has been named non-celiac gluten sensitivity (NCGS) (2-4).

It seems that NCGS is largely a self-reported diagnosis and would appear to be very common but reports have shown wide variations in prevalence (3-13%) (6). Its prevalence has been estimated to be six to ten-times higher than that of celiac disease (11).

The pathogenesis of this condition is remains unclear, roles of the innate immune system and intestinal permeability are the possibilities (12). Studies suggest that NCGS does not have a strong hereditary base, is not associated with malabsorption, and does not have an increased risk for long-term complications, such as autoimmune disorders or intestinal malignancy (7). However, natural history data on NCGS are still lacking. Therefore, it is difficult to draw firm conclusions on the outcome of this condition (15). It seems to be more frequent in adults than in children and more prevalent in females than in males (10). Numerous gastrointestinal symptoms such as Abdominal pain, bloating, nausea, diarrhea, constipation were reported with NCGS, which many of them suggested combination of IBS-like symptoms, (5) but it seems to be non- gastrointestinal symptoms (headache, fatigue, brain fog, joint and muscle pain, numbness in the hands and feet, depression, eczema and/or rash) that are more prevalent (4).

The diagnosis is based on exclusion of CD and wheat allergy in compatible clinical setting (symptoms associated with gluten contained diet and resolve with gluten free diet). This process involves detailed history-taking and negative immunoallergy tests to wheat by normal specific IgE; negative CD serology (anti-EMA and/or anti-tTG) in which IgA deficiency has been ruled out; normal duodenal histopathology because unlike CD, the small intestine biopsy in NCGS is usually characterized by normal mucosa or a mild increase in intraepithelial lymphocytes and patients show a resolution of symptoms when started on a GFD (12). If symptoms resolve with GFD, the patient should undergo a gluten challenge to confirm (13).

Therefore, it is possible to differentiate the three gluten-related conditions (WA, CD, and NCGS) based on a combination of clinical, biological, genetic and histological data.

Beyond the term of "non-celiac gluten sensitivity" new studies suggest that it may be possible that non-gluten proteins of wheat could be responsible for the associated symptoms such as wheat amylase trypsin inhibitor and lectin. Another possibility is a family of poorly absorbed dietary short-chain carbohydrates known as FODMAPs (fermentable oligo-, di-, and monosaccharides and polyols), which have osmotic effect and with their rapid fermentability, can lead to excessive fluid and gas accumulation and cause distention of the intestine, leading to functional GI symptoms. They found in a variety of foods, including those containing lactose, fructose, fructans, galactans, and polyols (sorbitol, mannitol, and xylitol) also, in wheat, rye, milk, some fruits (like apples and watermelons), vegetables (such as onions, garlic and asparagus) and legumes (like lentils and chickpeas) (14). Scientific evidence for these possibilities is lacking, and this remains a controversial topic (7,14). Since there is some degree of overlap between NCGS and other forms of wheat-exclusion responsive conditions (e.g., IBS responsive to low FODMAPs diet, non-IgE mediated WA), periodical patient reassessment (e.g., every 6–12 months), including an accurate dietary interview, is strongly recommended (13).

Gluten free diet is a main stay of treatment, which needs extensive education and referral to dietitian with expertise in social and emotional adaptation to living with food intolerances. Because many new problems could begin with this diet for example when patients removed gluten from their diet without proper substitutions, the possibility of nutritional deficiencies or their diet shifting to a low fiber, high fat type, which is harmful, is possible. In guiding patients, it is important to emphasize that it is not only about avoiding gluten, wheat, and/or high FODMAPs, but maintaining optimal nutritional intake and dietary habits over the long term (14,15).

Despite all of the above talks, there are still numerous questions about NCGS and many aspects of GS epidemiology, pathophysiology, clinical spectrum, and treatment and more research is needed to clarify this issue.
